# Sildenafil citrate long-term treatment effects on cardiovascular reactivity in a SHR experimental model of metabolic syndrome

**DOI:** 10.1371/journal.pone.0223914

**Published:** 2019-11-07

**Authors:** Yosra Doghri, Fabien Chetaneau, Moez Rhimi, Aicha Kriaa, Valérie Lalanne, Chantal Thorin, Emmanuelle Maguin, M. Yassine Mallem, Jean-Claude Desfontis

**Affiliations:** 1 UPSP NP3 (2017.B146), Nutrition, Pathophysiology and Pharmacology, Oniris, College of Veterinary Medicine, Food Sciences and Engineering, Atlanpôle—La Chantrerie, Route de Gachet, 5 BP, Nantes, France; 2 UMR 1319 Micalis, INRA, Microbiota Interaction with Human and Animal Team (MIHA), AgroParisTech, Université Paris-Saclay, Jouy-en-Josas, France; University of Mississippi Medical Center, UNITED STATES

## Abstract

Much evidence indicates that metabolic syndrome is strongly correlated with a decrease in nitric oxide and an increase in oxidative stress leading to cardiovascular alterations. In recent years, gut microbiota has emerged as a new contributor to the metabolic syndrome establishment and associated cardiovascular diseases, but the underlying mechanisms remain unclear. We hypothesized that a positive modulation of cyclic guanosine monophosphate (cGMP) pathway, through phosphodiesterase type 5 (PDE5) inhibition could prevent cardiovascular alterations and gut dysbiosis that may be associated to metabolic syndrome. Spontaneously hypertensive rats (SHR) were randomly divided into 4 groups: control, *cafeteria diet* (CD) and sildenafil citrate treated groups (5mg/kg per os) were given either a CD or a standard chow diet for 10 weeks. Body weight, arterial blood pressure and glucose tolerance test were monitored. At the 10^th^ week, cardiac inotropy and coronary perfusion pressure were evaluated on isolated heart according to Langendorff method. Cumulative concentration response curves to phenylephrine and acetylcholine were determined on thoracic aorta rings for vascular reactivity evaluation. Faecal samples were collected for the gut microbiota analysis. Compared to the control group, CD-fed rats showed a significant increase in body weight gain, arterial blood pressure and were glucose intolerant. This group showed also a decrease in β-adrenoceptor-induced cardiac inotropy and coronary vasodilation. Gut microbiota analysis revealed a significant reduction in the abundance of *Lactobocillus* spp in *cafeteria diet*-fed rats when compared to the control ones. Sildenafil citrate long-term treatment decreased weight gain and arterial blood pressure, improved coronary vasodilation and reduced α_1-_adrenoceptor-induced vasoconstriction in CD group. However, it did not reverse gut dysbiosis induced by chronic CD feeding. These results suggest that cGMP pathway targeting may be a potential therapeutic strategy for the management of the metabolic syndrome and associated cardiovascular disorders.

## Introduction

Metabolic syndrome is a complex systemic disorder characterized by a cluster of inter-related factors including abdominal obesity, abnormal glucose metabolism, hypertension, insulin resistance, endothelial dysfunction and inflammatory reaction [1,2]. It’s closely associated with the onset and development of cardiovascular diseases [3]. Recently, thermogenic, weight reducing and insulin sensitizing effects of phosphodiesterase 5 inhibitors have been reported, suggesting that a pharmacotherapy that elevates intracellular levels of cyclic guanosine monophosphate (cGMP) might be a promising approach to treat metabolic disorders [4,5]. Sildenafil citrate marketed as Viagra® is the first phosphodiesterase-5 inhibitor that has been widely used for the treatment of erectile dysfunction [6]. However, to the best of our knowledge, its metabolic and cardiovascular effects in an experimentally-induced metabolic syndrome in rats are not well known yet.

To date, recent studies have proposed the potential role of gut microbiota as a pathogenic factor affecting host metabolic balance and contributing to the development of metabolic syndrome [7,8]. Normal gut microbiota harbors the greatest density of microorganisms in the adult body [9] with *Firmicutes*, *Bacteroidetes* and *Actinobacteria* constituting the main dominant phyla [10]. It plays a crucial role to ensure the host homeostasis through several pathways including: Digestion and absorption of nutrients, gut permeability maintenance and intestinal immune system maturation [10,11]. Its composition is strongly influenced by diet as confirmed by studies conducted both on humans and animal models [12]. An altered gut microbiota composition has been demonstrated to cause devastating pathophysiological consequences such as obesity, metabolic disorders or type 2 diabetes [13]. However, the underlying mechanisms remain unclear until now.

Considering the intimate connection between intestine function and gut microbiota [14], the role played by cyclic nucleotides (i.e. cGMP) in the control of gut motricity, gut nutrient absorption and fluid-ion secretion [15], and the possible deregulation of these pathways that may occur under metabolic disorders [16], gut microbiota might be expected to undergo some changes following treatment with the nucleotide cyclic modulators.

Thus, the aim of this study is to evaluate the effects of chronic sildenafil citrate treatment on cardiovascular reactivity in spontaneously hypertensive rats (SHR) developing an experimentally-induced metabolic syndrome. We also attempted to analyze whether gut microbiota composition may be modified by sildenafil citrate chronic treatment, with the hypothesis that it could reverse the potential gut dysbiosis that may occur during metabolic syndrome.

## Materials and methods

### Animals

The experiments were performed in 32 male SHR (nine-week-old). All rats were obtained from Janvier Labs (Le Genest St Isle, France) and housed under a 12-hour light/ dark cycle at a constant temperature (22 ±1°C) and humidity (50%). Rats were acclimatized for one week before starting the experiments and were allowed access to standard chow and drinking water *ad libitum*.

All protocols used in this study were approved by the the Institutional care and Use Committee of Pays de La Loire, France (APAFIS N° 2884) and were carried out in strict accordance with the guidelines for the care and use of laboratory animals.

### Experimental procedures

Rats were randomly divided into 4 groups (n = 8 for each group): Control rats fed standard chow diet (4.5% total fat, 54.2% carbohydrates, 18.5% protein) (KLIBA NAFAG®, Kaiseraugst, Germany), Control SHR treated daily with Sildenafil citrate (5mg/Kg) [17] for 10 weeks by gastric gavage. The last 2 groups included *cafeteria diet- fed* (CD) group and CD–fed rats treated daily with Sildenafil citrate (5mg/Kg) for 10 weeks.

For *cafeteria diet* groups, rats were allowed to have free access to either *cafeteria diet* (6 different commercial chocolate, cookie and cereal bars, consisting of 18.2% total fat, 68.3% carbohydrates, 5.48% protein) or standard chow diet for 10 weeks. Rats received only one high calorie food per day that was switched daily to promote hyperphagia.

During the experimental protocol, body weight and abdominal circumference of all rats were monitored weekly.

### Arterial blood pressure measurement

Systolic and diastolic blood pressures were determined using the tail-cuff plethysmography, at Baseline, during and at the end of the study protocol. This method allows non-invasive measurement of the arterial blood pressure in the tail of conscious rats using volume pressure recording sensor technology (CODA®, Kent Scientific, USA).

### Glucose tolerance test

Before sacrifice, a glucose tolerance test was performed on fasted rats for 20 hours. The rats received an intraperitoneal injection of a glucose solution (1g/kg).

Blood samplings (one blood drop) were performed for glucose measurements (glucometer, Pura ®) on the rat’s tail vein before and 15, 30, 45, 60 and 90 minutes after the glucose loading. The area under the curve (AUC) was then calculated using Prism software (GrapPadPrism 5.0).

### Blood analysis

Blood samples were centrifuged at 5000g for 10 min at 4°C. Plasma was then collected and stored at -80°C until analysis. Total cholesterol and triglycerides were determined by automated enzymatic kits.

### Isolated heart preparation

At the 10^th^ week, rats were anesthetized with pentobarbital (54mg/kg i.p) and sacrificed by exsanguination of abdominal aorta. Hearts were quickly excised and put into a cold *Krebs-Henseleit* solution. The aorta was immediately cannulated and secured with a knot. The aortic cannula is connected with the Langendorff system allowing a retrograde perfusion of the heart at a constant flow rate of 12 ml min^-1^, with a *Krebs-Henseleit* solution previously filtered (0.2 μm filter funnel) and continuously oxygenated with 95% O2−5% CO_2_ gas mixture [18]. *Krebs-Henseileit* solution composition in mM: NaCl, 118.3; KCl, 4.7; MgSO_4_, 1.2; KH2PO_4_, 1.2; NaHCO_3_, 20; EDTA, 0.016; glucose, 11.1; and CaCl_2_, 2.5, (pH 7.4). Left ventricular pressure is measured via a water-filled latex balloon carefully inserted into the left ventricular after atrium incision. Both left venticular balloon and aortic cannula were connected to a pressure transducer. Perfusion pressure and left ventricular pressure were assessed using a PowerLab recorder and LabChart 7.0 software (ADInstruments). In order to assess the cardiac function, cumulative concentration- response curves to isoproterenol (a non- selective β-adrenergic agonist) were constructed (1ηM—1μM). Cardiac parameters have first been stabilized for 20 min with *Krebs-Henseleit* solution, then, the heart was perfused with each concentration of isoproterenol for 3 minutes. A washing with *Krebs-Henseleit* solution was done between each isoproterenol concentration. The heart’s contractile activity was assessed by analyzing the left ventricular developed pressure (LVDepP in mmHg), which was calculated as the difference between maximal systolic pressure and end-diastolic pressure whereas coronary vasodilation was assessed by analyzing perfusion pressure.

### Thoracic aorta preparation and vascular reactivity measurement

Descending thoracic aorta was rapidly isolated after exsanguination and placed in Krebs solution. The aorta is dissected, cleaned of fat and adherent connective tissue and cut into 3mm rings. Briefly, the rings were mounted in a 5ml organ bath containing *Krebs-Henseleit* thermostated at 37 ±0.5°C [19]. The isometric tension variation of each ring was detected by an isometric tension sensor (EMKA Technologies, Paris, France) and recorded by data Acquisition software (Acknowledge 4.1, BIOPAC system, MP 150, CEROM, Paris France). After a 60-min of equilibration at a resting tension of 2g, the viability of the endothelium of the control group was confirmed by obtaining at least 60% relaxation to acetylcholine (1 μM) in rings that had already been pre-contracted with phenylephrine (1μM), a selective α1-adrenoceptor agonist. In order to assess vascular reactivity, cumulative concentration-response curves (CCRC) to phenylephrine (1ηM– 10μM) have been performed. After a wash out and reequilibration, rings were preconstructed to 80% of the maximal phenylephrine-induced contraction. Once the contraction reached a plateau, a cumulative concentration–response curve to acetylcholine (1ηM—10μM) was then constructed.

### Determination of cGMP production in thoracic aortic rings and epididymal fat

Immediately after the rats’ sacrifice, thoracic aorta rings and epididymal fat samples were frozen in liquid nitrogen to prevent cGMP degradation and were kept at -80°C. The samples were then treated with 6% cold trichloroacetic acid and centrifuged at 1500g for 10 min at 4°C. The supernatant fractions were extracted three times with water saturated diethyl ether (5 volumes of diethyl ether to 1 volume of supernatant). Residual diethyl ether was evaporated by heating samples to 70°C for 5 min. The dried extract was dissolved in an assay buffer and the cGMP concentrations were measured colorimetrically by use of an immunoenzymatic assay kit (Cayman Chemical Company). Absorbance was read on a spectrophotometer at 405nm. The mean value was calculated from duplicate measurements of each sample and normalized to total cell protein levels previously measured using a protein assay reagent kit (micro BCA-Pierce) [20].

### Gut microbiota analysis

Fecal samples were taken before and at the end of the experimental protocol from all rats. The samples were frozen in liquid nitrogen and stored at a temperature of -80°C.

The V3-V4 region was amplified from purified DNA with the primers F343 (CTTTCCCTACACGACGCTCTTCCGATCTACGGRAGGCAGCAG) and R784 (GGAGTTCAGACGTGTGCTCTTCCGATCTTACCAGGGTATCTAATCCT) using 30 amplification cycles with an annealing temperature of 65°C. The amplicon lengths were about 510 bp (the exact length varies depending on the species). Because MiSeq sequencing enables paired 250-bp reads, the ends of each read overlap and can be stitched together to generate extremelyhigh-quality, full-length reads covering the entire V3-V4 region. Single multiplexing was performed using a home-made 6 bp index, which was added to the R784 primer during a second PCR with 12 cycles using the forward primer (AATGATACGGCGACCACCGAGATCTACACTCTTTCCCTACACGAC) and the modified reverse primer (CAAGCAGAAGACGGCATACGAGAT-index-GTGACTGGAGTTCAGACGTGT). The resulting PCR products were purified and loaded onto the Illumina MiSeq cartridge according to the manufacturer instructions. The quality of the run was checked internally using PhiX, and for further analysis, each pair-end sequence was assigned to its sample using the previously integrated index.

## Drugs

Pentobarbital solution was purchased from CEVA santé animale (France); phenylephrine hydrochloride, acetylcholine chloride and isoproterenol were provided by Sigma Aldrich Chimie® (Saint Quentin-Fallavier, France), sildenafil citrate (Sandoz®) is a human medicine.

All drugs were prepared as stock solutions in distilled water.

### Statistical analysis

Results were expressed as a mean ± SEM of n experiments, where n is the number of rats. Statistical analysis was performed using two-way analysis of variance for multiple group comparisons followed by Tukey post-hoc test where appropriate.

Measurement of body weight, abdominal circumference, arterial blood pressure and data from isolated perfused heart were evaluated by a linear mixed effect model (LME) using R software. Relaxation was expressed as the percentage relaxation of the phenylephrine-induced precontraction. Different CCRCs and their parameters (maximum effect: E_max_ and the negative logarithm of the concentration producing 50% of the maximum effect or pD_2_) were compared using a non-linear mixed effect model using R software (NLME) [21]. All graphs have been performed using (PRISM® software version 5).

Microbiome statistical analysis following rarefaction of all communities to even sampling depths, the abundances of all families were computed by agglomerating the OTUs assigned to those families. For each comparison between all groups, the difference of the microbiota composition at phylum, family and genus level were assessed using kruskal-wallis test with BH correction [22] for multiple comparisons.

*P*<0.05 was considered to indicate a statistically significant difference.

## Results

### Weight gain, arterial blood pressure, glucose tolerance and blood analysis

**Fig 1:**
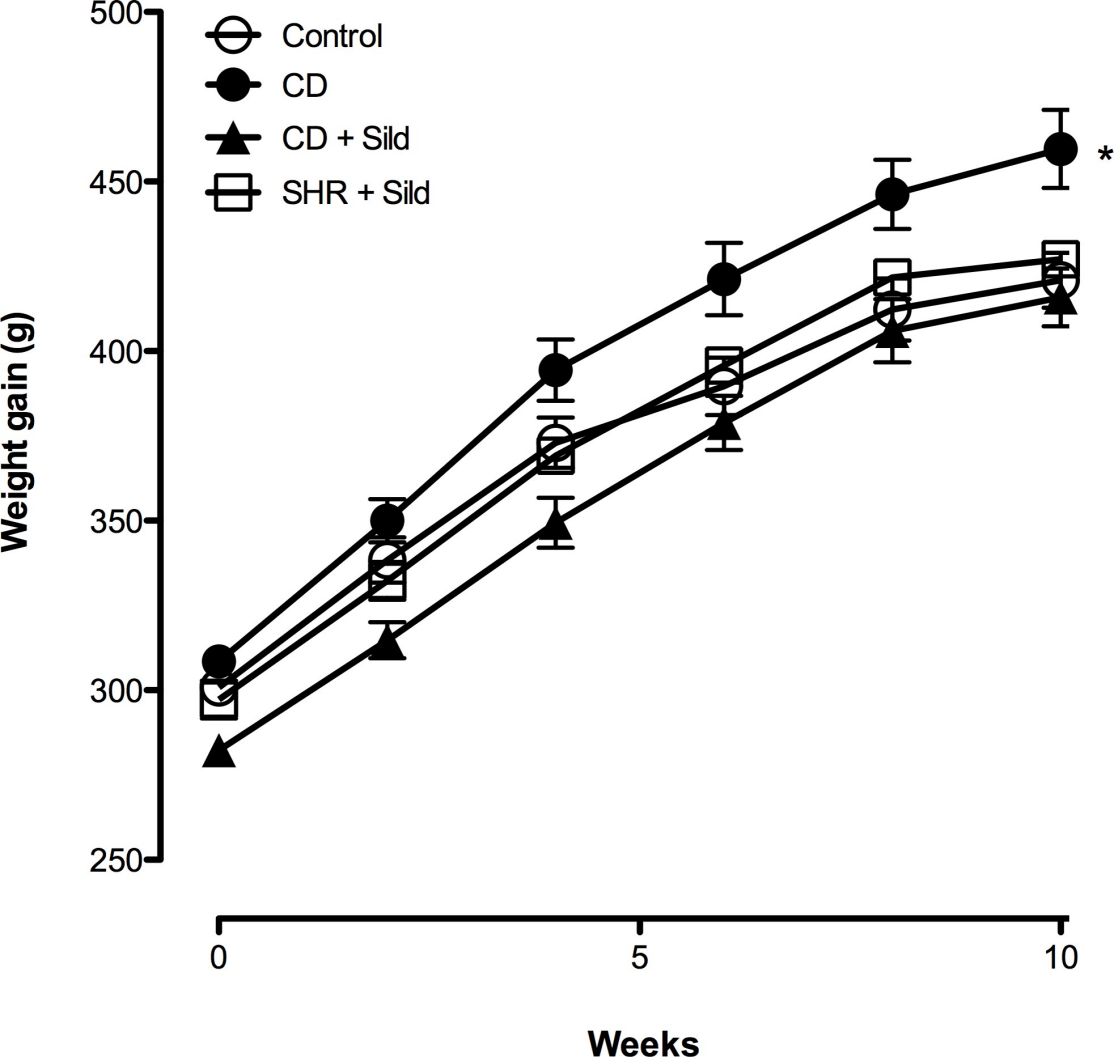
Effect of *Cafeteria* diet (CD) and long-term treatment with Sildenafil Citrate (Sild) on body weight. Compared to standard diet-fed rats, CD-fed group had a higher weight gain. Sildenafil citrate treatment reduced excessive weight gain. Data are represented as mean ±SEM. *p< 0.05 *Vs* control group using a LME model.

Within 10 weeks, CD feeding significantly increased the body weight of CD fed rats compared to the standard chow diet-fed group (151.3 ±11.4g *Vs* 120.0 ±5.7g) (Fig 1). This weight gain was associated with a significant increase in abdominal circumference and epididymal fat mass. Chronic sildenafil citrate treatment resulted in lower weight gain compared with untreated CD- fed rats (Fig 1).

**Fig 2:**
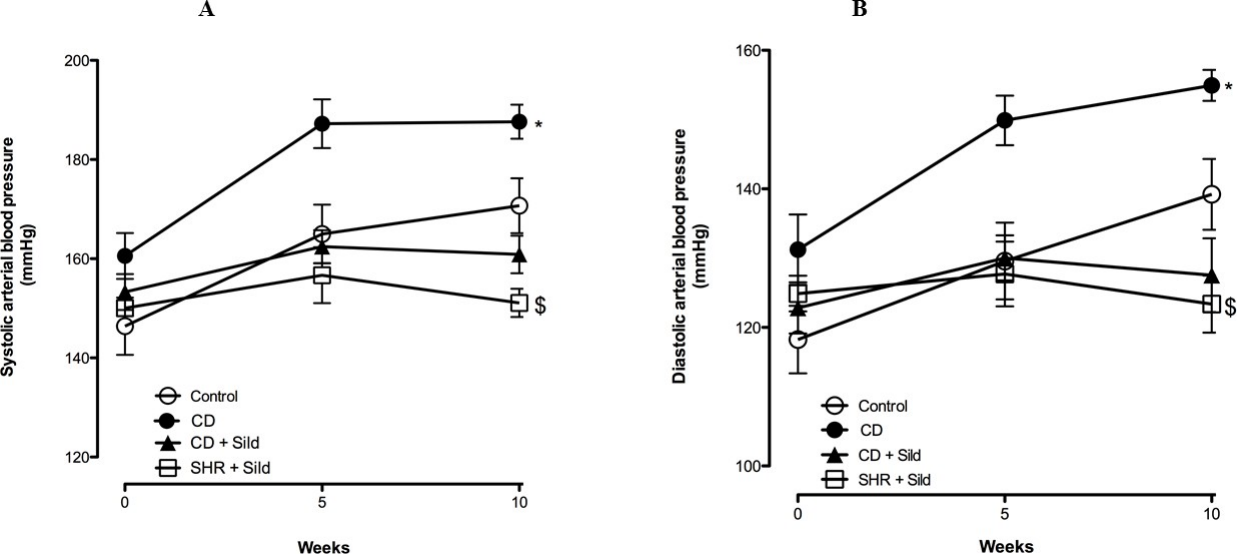
Effect of *Cafeteria* diet (CD) and long-term treatment with Sildenafil Citrate (Sild) on arterial blood pressure. Rats fed CD for 10 weeks showed increased systolic arterial blood pressure **(A)** and diastolic blood pressure **(B)** when compared to rats fed a standard diet. Sildenafil citrate chronic treatment significantly reduced blood pressure in both control and CD group. Data are represented as mean ±SEM. *p< 0.05 *Vs* control or CD + Sild $ *p*<0.05 *Vs* control compared by a LME model.

CD-fed rats also presented elevated arterial systolic and diastolic blood pressure at the end point of the experiment (187.63 ±3.42 mmHg *Vs* 170.68 ±5.53 mmHg, 154.93 ±2.23 mmHg *Vs* 137.96 ±5.80 arterial systolic and diastolic blood pressure in CD- fed group and control group respectively). Long-term treatment with sildenafil citrate significantly reduced blood pressure in both control and CD group. However, the decrease in sildenafil-induced arterial blood pressure was greater in the CD group than in the control group (Fig 2A and 2B).

**Fig 3:**
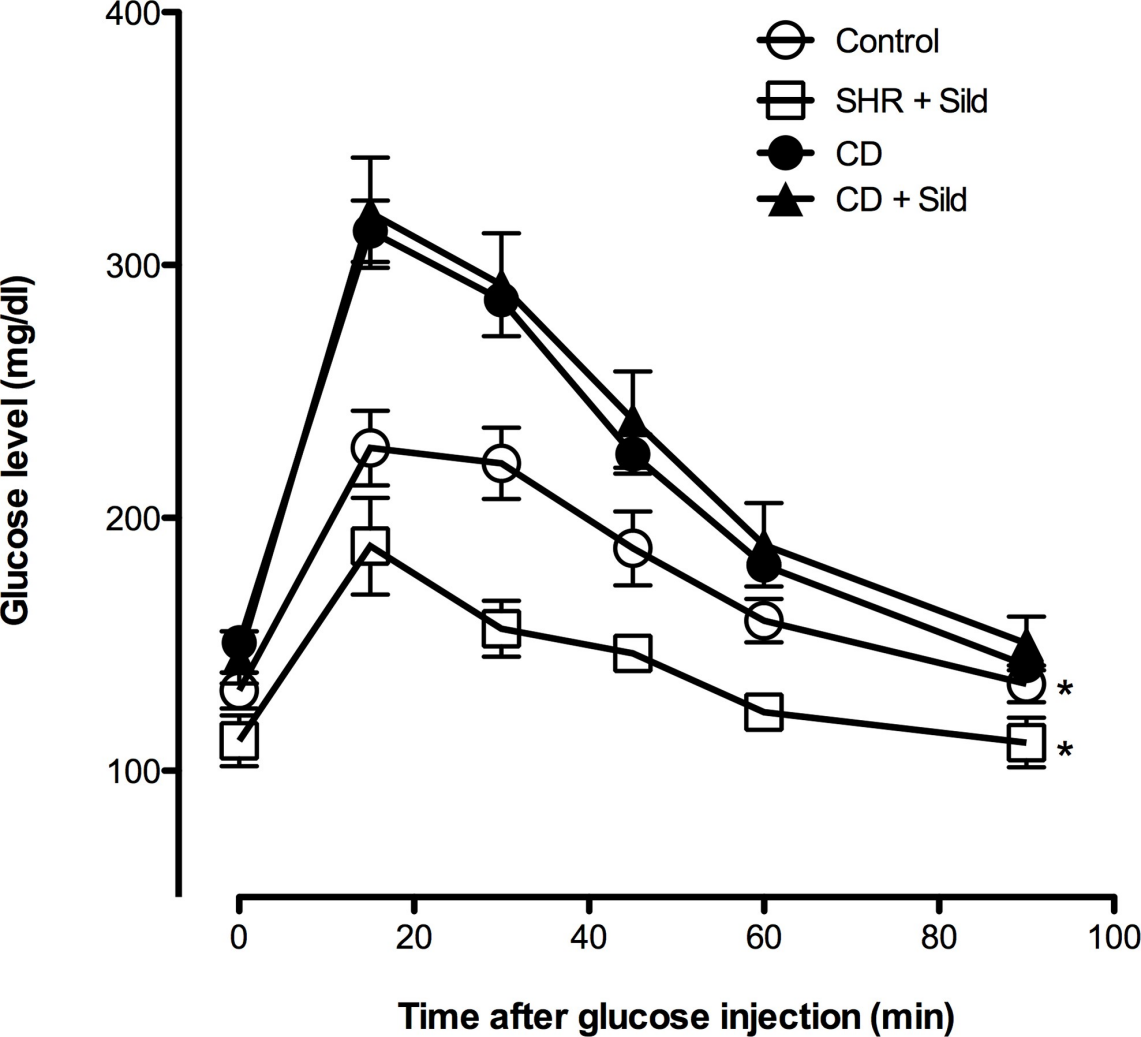
Effects of *cafeteria-diet* feeding and sildenafil citrate treatment on blood glucose concentrations during glucose tolerance test. Blood glucose concentration measured during an intraperitoneal glucose tolerance test performed on fasted rats at the end of the 10^th^ week of the protocol. AUC compared with CD group by two-way ANOVA followed by Tukey’s Post hoc test *p< 0.05.

Chronic cafeteria diet feeding showed enhanced elevation of blood glucose during glucose tolerance tests in comparison with control group (Fig 3). The increase reached its peak after 15 min followed by a delayed and slow decrease. On the other hand, sildenafil citrate long-term treatment significantly reduced fasting blood glucose (150.6 ± 6.78 mg/dl *Vs* 111.8± 9.98 mg/dl in CD group and Sild + SHR respectively) and improved glucose intolerance only in the control group (Fig 3).

Blood analysis revealed that CD-fed rats had elevated fasting glucose level when compared to SHR sildenafil citrate treated ones with unchanged total cholesterol and triglyceride levels (Table 1).

**Table 1:** Blood analysis.

	Control	SHR + Sild	CD	CD + Sild
Blood glucose (mg/ dl)	131.8±7.17	111.8±9.98 *	150.6±6.78	144.9±10.3
Total cholesterol (g/l)	0.99±0.04	0.83±0.05	0.89±0.02	0.89± 0.017
Triglyceride (g/l)	0.896±0.11	0.86-±0.08	0.947± 0.06	1.038±0.1

Fasting blood glucose, plasmatic total cholesterol and triglyceride measured at the end of the protocol. Data are expressed as mean ±SEM.

*p< 0.05 compared with the CD group by two-way ANOVA test followed by Tukey’s Post hoc test when appropriate.

### ß adrenoceptor stimulation on isolated perfused heart

To completely eliminate the effects of *in vivo* regulatory mechanisms on cardiac function, perfused hearts on Langendorff apparatus were used to examine intrinsic cardiac contractility and coronary perfusion pressure at baseline and after increasing concentrations of isoproterenol stimulation *ex vivo*. At baseline, cardiac contractility determined by LDevP, was significantly reduced in CD-fed rats when compared to the control ones (p = 0.003) (Table 2). On the other hand, long-term treatment with sildenafil citrate restored this parameter to control values.

**Table 2:** Basal values of cardiac inotropy and coronary perfusion pressure.

	**Control**	**CD**	**CD + Sild**	**SHR + Sild**
LVDevP (mmHg)	78.27 ± 5.83 *	48.08 ± 5.38	73.79 ± 4.69 *	56.25± 2.006
Coronory perfusion pressure (mmHg)	31.43 ± 2.41	27.23 ± 3.22	31.98 ± 3.61	35.87± 3.12

Data are represented as mean ±SEM. *p< 0.05 *Vs* CD

**Fig 4:**
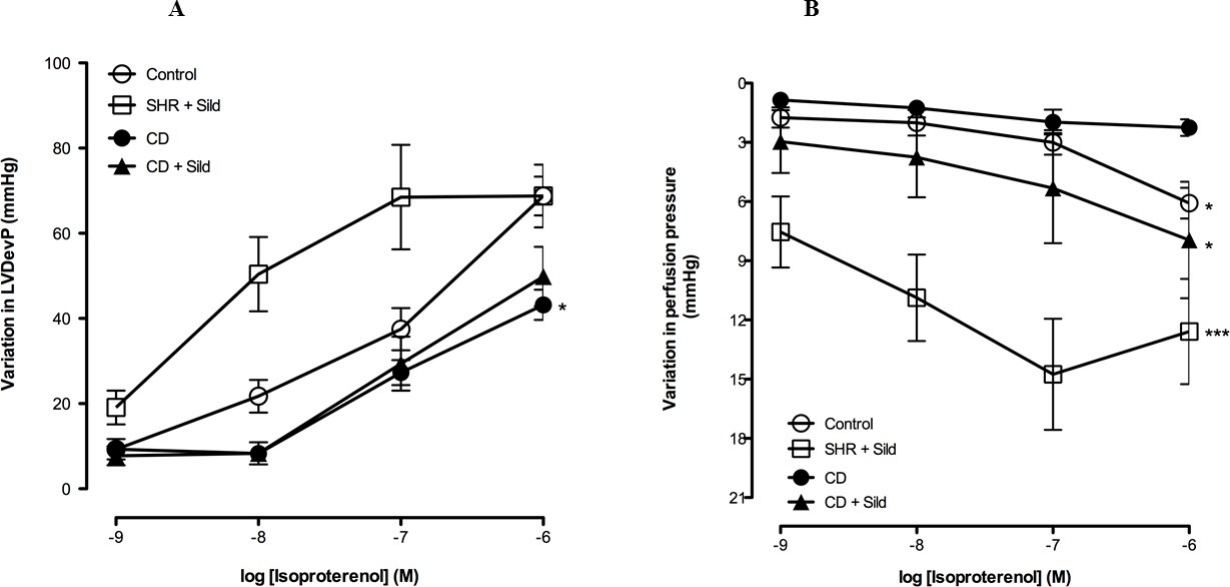
Effects of *cafeteria-diet* feeding and sildenafil citrate treatment on cardiac function. Cardiac inotropy **(A),** coronary vasodilatation **(B)**. Compared to control rats, CD-fed rats showed reduced cardiac inotropy and coronary vasodilation. Sildenafil citrate treatment significantly improved cardiac inotropy and coronary vasodilation in control SHR but only improved coronary vasodilation in CD-fed rats. LVDevP: left ventricular developed pressure, CD: *Cafeteria diet*. Values are expressed as mean ±SEM. (A) *p< 0.05 CD *Vs* Control, SHR+Sild **(B)** *p< 0.05 CD *Vs* Control, CD + Sild, *** *p*< 0.001 CD *Vs* SHR + Sild.

To determine the effects of ß-adrenoceptor stimulation on cardiac contractility, different concentrations of isoproterenol were applied to the hearts. There were concentration-dependent increases of cardiac inotropy in all groups. However, the isoproterenol-induced positive inotropic effect was significantly attenuated in CD-fed rats (p = 0.009) compared to the control SHR group (Fig 4A) suggesting an alteration of the ß-adrenergic response in this group.

On the other hand, isoproterenol induced a concentration-dependent coronary vasodilation in all groups. This effect was significantly reduced in CD-fed SHR compared to the control group (p = 0.017) (Fig 4B).

Sildenafil citrate long-term treatment significantly improved cardiac positive inotropy (p = 0.016) and coronary vasodilation (p<0.001) in control SHR in comparison with untreated CD group, but only improved coronary vasodilation in CD-fed group (p = 0.026) (Fig 4).

### Vascular reactivity

**Fig 5:**
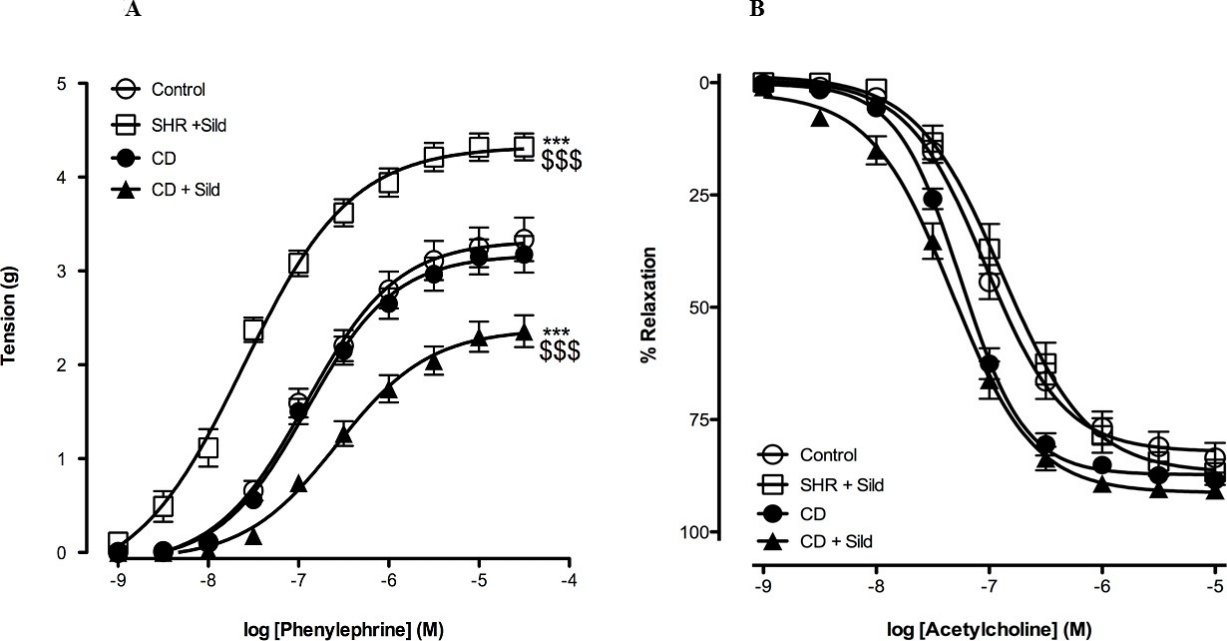
Effect of *cafeteria diet* feeding and sildenafil citrate treatment on vascular reactivity. Cumulative concentration response curve (CCRC) to phenylephrine in thoracic aortic rings isolated from all groups **(A)**, Concentration-response curves to acetylcholine in thoracic aortic rings precontracted with phenylephrine **(B).** Data are represented as mean ±SEM. *** *p*< 0.001 *Vs* Control / CD group for Emax. $ $ $ *p*<0.001 *Vs* Control / CD group for pD_2_ compared by NLME model.

In this study, we examined the effect of sildenafil citrate chronic treatment on the aortic contractile response to phenylephrine, a selective α_1_-AR agonist. The CCRC to phenylephrine obtained in isolated aortic rings of CD group treated with sildenafil citrate was significantly shifted to the right and the bottom when compared to the control rats (Fig 5A; Table 3). Surprisingly, phenylephrine-induced maximal contractile response (E_max_) and pD_2_ value were significantly higher in aortic rings isolated from SHR treated with sildenafil citrate than control rats (Table 3, Fig 5A). However, there was no change neither in the maximal contractile response nor in pD_2_ value between control and CD group.

**Table 3:** pD_2_ and E_max_ of phenylephrine responses in aortic rings isolated from all groups.

	Control	SHR + Sild	CD	CD + Sild
E_max_	3.27±0.21	4.17±0.16 ***	3.14±0.18	2.35±0.17 ***
pD_2_	6.87±0.04	7.69±0.09 ***	6.84±0.04	6.54±0.06 ***

Values are means ±SEM.

***p <0.0001 compared with the control group by NLME model

The cumulative dose response curves to acetylcholine-induced endothelium-dependent vasorelaxation are shown in (Fig 5B). Vasorelaxation response to acetylcholine did not show any significant difference either by *cafeteria diet* feeding or sildenafil citrate treatment, indicating that vasodilator action of acetylcholine was not impaired in thoracic aortic rings SHR. However, the maximal relaxation in response to acetylcholine was slightly higher in CD-fed rats treated with sildenafil citrate.

### cGMP levels

cGMP intracellular concentrations were determined in thoracic aortic rings. There was a slight decrease in cGMP levels but not statistically significant, in both thoracic aorta and epididymal fat samples, in *Cafeteria diet*- fed group compared to the control group. No difference was observed in sildenafil citrate treated rats either (Table 4).

**Table 4:** Intracellular cGMP levels in thoracic aortic rings and epididymal fat samples.

	**cGMP (pmol/mg protein)** **Thoracic aorta**	**cGMP (pmol/mg protein)** **Epididymal fat**
Control group	15.67 ±2.26	1.86 ±0.65
CD-fed group	11.04 ±2.30	0.86 ± 0.17
CD group treated with sildenafil citrate	9.63 ± 1.45	1.22 ± 0.19

Data are represented as mean ±SEM

### Gut microbiota analysis

**Fig 6:**
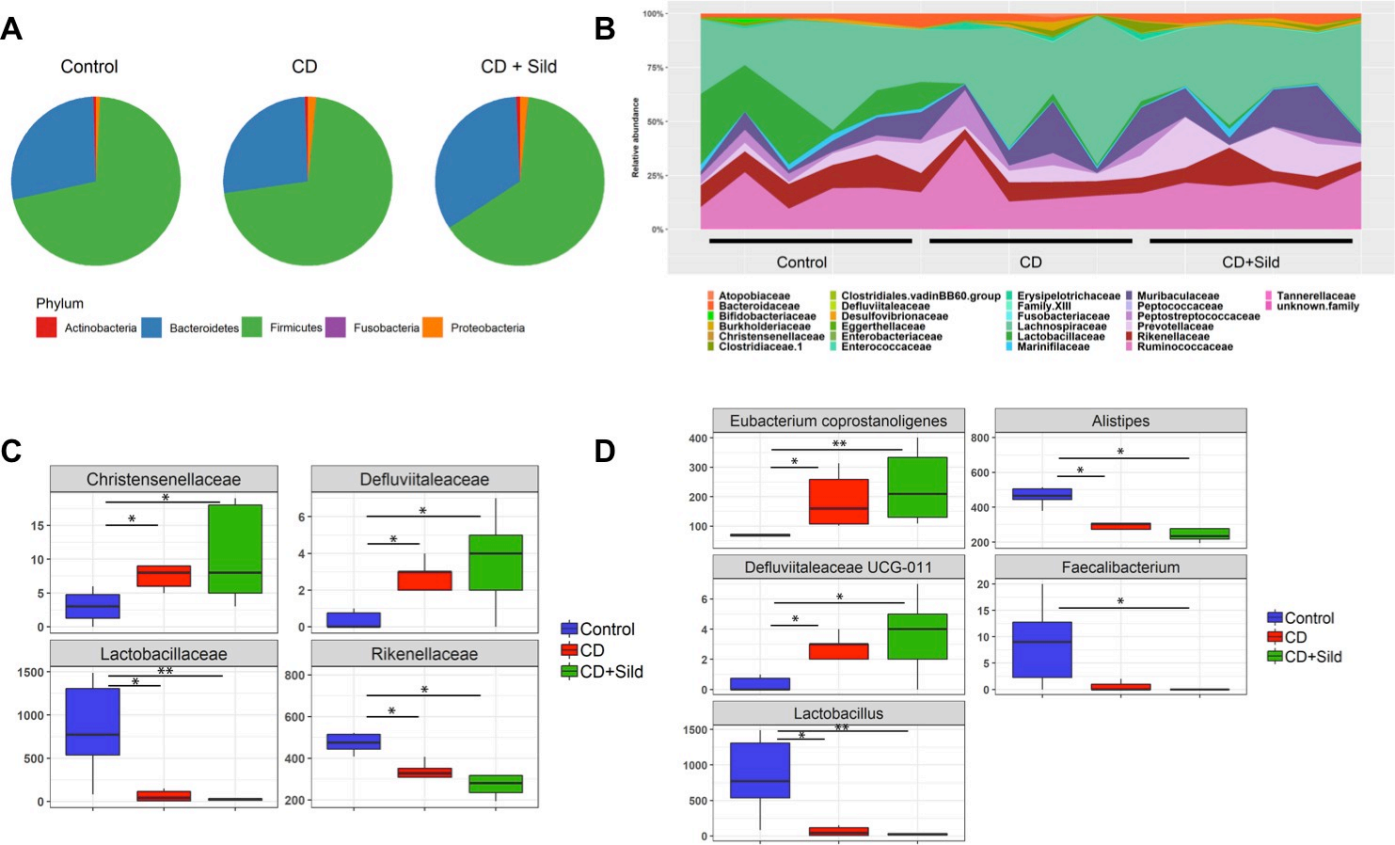
Gut bacterial community analysis by 16S rRNA gene high-throughput sequencing. (A) Composition of abundant bacterial phyla identified in the microbiota of the three different groups. (B-C) Different bacterial families in each sample among Control, CD and CD+Sild groups. (D) Various bacterial genera in each sample among each group of rats. Data are presented as box plots. ‘*’ and ‘**’ indicate a significant difference (P<0.05 and P<0.01 respectively).

Several studies performed on animals and humans have reported a link between gut microbiota composition and components of the metabolic syndrome. In our study, gut microbiota analysis revealed the presence of the main phyla such us *Firmicutes*, *Bacteroidetes*, *Actinobacteria* and *Proteobacteria* in all rats. Thus, *cafeteria diet* feeding for 10 weeks did not alter the gut communities at the phylum level. However, we observed significant differences in the abundance of some bacterial families. The gut microbiota analysis showed that *cafeteria die*t-fed rats had less abundance of *Rikenellaceae* (*p* = 0.029) and *Lactobacillaceae* (*p* = 0.019) than control SHR. On the other hand, the sildenafil citrate long-term treatment did not resotre gut dysbiosis unduced by chronic cafeteria diet feeding (Fig 6).

## Discussion

In the present study, we showed that 10 weeks of CD induced metabolic syndrome in rats by causing abdominal obesity, glucose intolerance and arterial hypertension. These results are consistent with the findings of Miesel et al. [23] who demonstrated that *cafeteria diet*-fed SHRs feature arterial hypertension, obesity and an altered glucose levels in response to the oral glucose tolerance test (OGTT), mimicking the human metabolic syndrome. CD consists mainly of a palatable diet with a more balanced caloric composition that resembles a Western diet. These disorders are also found in other studies of diet-induced metabolic syndrome models [24]. Miesel et al. [23] showed that SHR became hyperlipidemic when fed with *cafeteria diet*. Our findings regarding the cholesterol and triglyceride levels of SHR were in consistence with the results of Rehakova et al. [25]. However, in our study, rats did not develop dyslipidemia over the feeding periods. It has already been shown that SHR generally do not develop hypercholesterolemia and hyperlipidemia unless they are exposed to a special diet such us high-cholesterol or high-fructose high-fat diet [26]. The absence of dyslipidemia in rats despite the high caloric diet could well be due to the fact that cholesterol transport through the vascular wall and reverse cholesterol transport is so efficient in rats that it prevents the development of severe hypercholesterolemia [27].

Sildenafil citrate long-term treatment reduced body weight gain and visceral fat in CD-fed rats. The present results are consistent with those of Ayala et al. [4] who showed that treatment with sildenafil for 12 weeks prevented excessive weight gain in high fat-fed mice by preventing diet-induced energy imbalance. Moreover, Ryu et al. [28] showed that PDE5 inhibitor treatment was able to suppress inflammatory markers expression that play a critical role in the induction of obesity. Thus, long-term sildenafil citrate treatment may reduce weight gain by different mechanisms than fat cell lipolysis. This would explain epididymal fat cGMP levels not significantly different between untreated and sildenafil citrate-treated rats.

Even though the CD did not induce fasting hyperglycemia in our experimental conditions, the CD-fed group presented impaired glucose tolerance as previously reported [29]. Although 10 weeks of chronic sildenafil citrate treatment significantly reduced weight gain and abdominal obesity in the CD-fed SHR, the impairment of glucose tolerance was not attenuated. It has been shown in previous studies that long-term PDE5 inhibition plays a role in countering the effects of high-fat diet-induced insulin resistance and improving pancreatic β-cell function as assessed by oral glucose tolerance testing [4,30]. However, almost all studies were either conducted on lean animals or before the onset of insulin resistance which could explain the lack of glucose intolerance improvement observed in our study. The resulting effects of sildenafil citrate treatment may differ between healthy and pathological states [5]. Our results support this hypothesis since sildenafil citrate chronic treatment significantly restored glucose metabolism in standard chow diet-fed rats.

It is already well known that obesity is characterized by sympathetic nervous activation [31] which in turn may induce β-adrenergic receptor dysregulation [32]. In the present study, we aimed to compare the effects of β-adrenoceptor stimulation in SHR, CD-fed rats and in sildenafil citrate treated rats that were exposed to either CD or standard chow diet, using Langendorff method on isolated heart. We precisely assessed the effects of β-adrenergic stimulation on cardiac inotropy and coronary arteries vasodilation through LVDevP evaluation and perfusion pressure variations respectively. Our data showed a significant decrease in the positive inotropic effect of β-adrenoceptor stimulation as well as a significant decrease in coronary vasodilation in CD-fed rats. The mechanisms underlying impaired β-responsiveness remain unclear. It’s not well known whether this attenuated response to isoproterenol stimulation is due to decreased expression of β-adrenoceptors [33], to altered β-adrenergic signaling pathways or to impaired Ca^2+^ handling [34,35]. Only complementary studies could allow to better understand these divergent findings.

In the current study, we did not show any change neither in cardiac inotropy nor in coronary vasodilation between SHR group treated with sildenafil citrate and control group. However, we showed that chronic sildenafil citrate treatment significantly restored coronary vasodilation, and slightly although not significantly ameliorated cardiac inotropy in CD group suggesting that chronic PDE5 inhibition was able to prevent cardiac alteration due to the metabolic syndrome.

Several studies have previously demonstrated that long-term PDE5 inhibition resulted in a lower adrenergic vasoconstrictive response of the vascular bed [36]. Unexpectedly, the present results showed a leftward shift of the phenylephrine curve in aorta from sildenafil citrate-treated SHRs. According to the work of Teixeira-da-Silva et al.[36], treatment with sildenafil citrate reduced phenylephrine-induced contraction in aortic rings taken from SHR. However, in that study, the authors used the dose of 45mg/kg for 60 days which is 9 times higher than that used in our study. Moreover, all rats were treated before the onset of the hypertensive state while rats included in our protocol were hypertensive from the start of the experiments. The increase in E_max_ and pD_2_ values of phenylephrine in aortas from sildenafil citrate-treated SHR may suggest an increased α_1_-AR density and /or sensitivity that was likely developed to offset the effect of prolonged PDE5 inhibition. Interestingly, we found that the phenylephrine-induced vasoconstriction was reduced by sildenafil citrate treatment in CD-fed group, suggesting that compensatory mechanisms linked to metabolic syndrome might have been developed in CD-treated rats to limit the phenylephrine hyper-responsiveness observed in control SHRs. From these results, it can be suggested that PDE5 inhibition, under our experimental conditions, plays a functional role by modulating aortic reactivity probably *via* a change in cGMP intracellular level. Since sildenafil citrate treatment is expected to increase intracellular cGMP level, it was reasonable to compare tissular aortic cGMP content between untreated and treated rats with sildenafil citrate. Our results showed vascular cGMP levels in the same range as those reported in previous studies [37]. But unexpectedly, we failed to detect any significant accumulation of vascular cGMP in aortic rings from sildenafil citrate-treated rats. This result suggests that the change of aortic rings response to phenylephrine observed in our study may result from other mechanisms of action than direct inhibition of PDE5, as described elsewhere, PDE5 inhibition, during sildenafil citrate treatment has coordinated stimulatory action on both cAMP and cGMP signaling pathways [38].

In the present work we showed that sildenafil citrate prevented the development of arterial hypertension in both control-treated and CD-treated group. Although the mechanism involved in this finding has not been specifically addressed, reduction in vascular resistance could be considered as a contributing mechanism accouting for the ability of sildenafil citrate to limit the developpement of arterial hypertension. Proximal resistance vessels such as the mesenteric arteries, contribute substantially to the peripheral resistance and play an important role in the maintenance of arterial pressure. It has already been shown that both PDE5 expression and activity were significantly higher in mesenteric arteries in SHR rats when compared to normotensive rats, and that sildenafil citrate was able to suppress PDE5 activity in these arteries [39].

Interestingly we found that the prevention of the time-dependent increase of arterial hypertension by sildenafil citrate treatment was more marked in the CD group than in the control one. The reason of this discrepancy is not readily apparent. The extent of drugs-induced antihypertensive effect has been reported to closely depend upon the level of blood pressure [40,41]. Thus, it is not unlikely to postulate that differences in sildenafil citrate effect observed in our study might be due to the level of blood pressure and/or the pre-existing level of cardiovascular alterations (*i*.*e*. *increase of sympathetic tone*) that could have been developed during the metabolic syndrome in CD group. We cannot however rule out the possibility that the difference observed between control-treated and CD-treated group could involve the ability of sildenafil citrate to reduce blood pressure by additional mechanisms that may occur independently of its vascular effects. In support of this assumption, sildenafil was reported to exert its effect on blood pressure by reducing angiotensin II levels and restoring the baroreflex sensitivity [42].

Conversely to previous report describing a decreased NO bioavailability in vascular wall of adult SHR [43], our study did not reveal any alteration in acetylcholine-induced vasorelaxation neither in control rats nor in CD-fed rats. We did not observe significant change in intracellular cGMP levels between the different groups as previously mentioned, suggesting that CD feeding did not induce endothelial dysfunction in SHR aorta. The lack of impairment of the endothelium-dependent relaxation observed in our experimental conditions, does not necessarily confirm the absence of endothelial dysfunction. It may be possible that rats were only in a very stage of the pathology development and that duration of the diet may not be severe enough to exhibit the impaired response to acetylcholine [44]. Nonetheless, Berenyiova et al. [45] showed that SHR develop an adaptive mechanism to preserve NO-dependent vasorelaxation and NOS activity level.

Gut microbiota is emerging during the last decade, as a new major contributor to the development of metabolic syndrome. Different studies have already shown that diet-induced metabolic syndrome is associated with decreased gut microbiota diversity and richness. In this context, several studies have reported that *cafeteria diet* feeding induces large gut microbiota changes with a significant reduction in *Bacteroidetes* and a corresponding increase in *Firmicutes* [46,47]. The shift in the relative abundance in these phyla has been proposed as linked to the increased capacity to harvest energy from food and with increased low-grade inflammation [48]. Conversely to those findings, our data did not reveal marked changes in the gut microbiota composition at the phylum level after chronic CD feeding. These discrepancies could be attributed to the difference in the strain of rats used. In fact, in the previously mentioned studies, the experiments were conducted on normotensive rats whereas in our study we worked on the SHR. In this regard, Yang et al. [49] has reported a significant decrease in gut microbiota diversity, richness and evenness in SHR when compared to normotensive rats, in addition to an increased *Firmicutes* to *Bacteroidetes* ratio. That study has also shown a decrease in acetate- and butyrate- producing bacteria in SHR. Therefore, the preexistence of a gut dysbiosis in the SHR may hide the impact of the CD on the gut micorbiota composition at the phylum level. Nonetheless, other studies have found no differences either between *Firmicutes* and *Bacteroidetes* at the phylum level [48]. However, our study showed significant differences at the family and species level between groups. Indeed, we observed a remarkable decrease in *Lactobacillus* spp in *cafeteria diet*-fed rats when compared to control ones. These results are consistent with findings of Lecomte et al. [50] who showed a reduction in the abundance of the *Lactobacillus* species in a rat model of metabolic syndrome. This reduced abundance of *Lactobacillus* species has been shown to be negatively correlated with fat mass and body weight. Moreover, according to Lecomte et al. [50], lactobacilli play a critical role in preserving intestinal barrier integrity through maintenance of cell-to-cell junctions. Thus, a lower abundance of this species is assumed to promote the passage of endotoxins such us lipopolysaccharides into the bloodstream increasing the proinflammatory molecules release and maintaining insulin resistance [51]. Furthermore, it has been reported in previous study that long-term ingestion of *Lactobacillus* spp was able to enhance lipolysis and to reduce body weight as well as abdominal fat weight [52]. Collectively, these findings may involve a role of *Lactobacillus* spp in promoting the metabolic syndrome development. To the best of our knowledge, our study is the first to investigate the effects of sildenafil citrate long-term treatment on gut microbiota composition. Although, long-term sildenafil citrate treatment prevented excessive weight gain in CD-fed rats, it did not reverse gut dysbiosis induced by chronic CD feeding. Yet, it has been shown that cGMP is considered as a key factor in regulating the immune function in the intestine and in maintaining the intestinal barrier integrity [15]. It would be likely that the duration of sildenafil citrate treatment may not have been sufficient to modify gut microbiota composition of treated rats. Further studies are necessary to better understand the interaction between gut microbiota and sildenafil citrate treatment.

In conclusion, the current study demonstrated that CD feeding induced metabolic abnormalities, an impairment of cardiac contractility and decreased several bacterial species of the gut microbiota, especially *Lactobacillus* spp. in SHR. Chronic treatment with sildenafil citrate showed metabolic protective effects in our rat model of metabolic syndrome. However, its beneficial effects on cardiovascular activity are less evident. The potential of PDE5 inhibition as a pharmacotherapeutic option in the treatment of metabolic syndrome warrants further investigation.
